# The Relationship Between Race and Gestational Diabetes Mellitus

**DOI:** 10.7759/cureus.92941

**Published:** 2025-09-22

**Authors:** Smita Saji, Sakhi Shah, Sarah Stumbar, Pura Rodriguez de la Vega, Juan Lozano

**Affiliations:** 1 College of Medicine, Florida International University Herbert Wertheim College of Medicine, Miami, USA; 2 Family Medicine, Florida International University Herbert Wertheim College of Medicine, Miami, USA; 3 Medical and Population Health Sciences Research, Florida International University Herbert Wertheim College of Medicine, Miami, USA; 4 Translational Medicine, Florida International University Herbert Wertheim College of Medicine, Miami, USA

**Keywords:** gestational diabetes, health disparities, maternal outcomes, pregnancy, racial disparities

## Abstract

Introduction

Gestational diabetes mellitus (GDM) is diabetes that is newly developed by women during pregnancy. There is an existing gap in the literature in the United States on the relationship between race and GDM; the results of this study will seek to address this gap by examining pregnant women of color in comparison to their White, non-Hispanic (NH) counterparts. Given the potential health risks posed by GDM and the overall impact of the social determinants of health, it is essential to address racial disparities in GDM.

Methods

A retrospective cohort study was conducted using data representative of the United States, which was acquired from the National Vital Statistics System (NVSS) from the National Center for Health Statistics (NCHS). The racial groups investigated included NH White, NH Black, NH American Indian or Alaskan Native/Native Hawaiian or Other Pacific Islander (AIAN/NHOPI), NH Asian, NH Mixed, and Hispanic women, while the outcome of interest was diagnosis of GDM. Initially, the sample's baseline characteristics were evaluated, followed by a bivariate analysis to ascertain any association between the exposure and outcome, as well as to pinpoint potential confounders. Ultimately, we conducted a multivariable analysis to control for confounders.

Results

A total of 3,367,601 women were included in our study. Most women included were NH White women (50.5%), followed by Hispanic women (26.5%), NH Black women (13.5%), and NH Asian women (6.1%). The adjusted odds of GDM were more than twice as high in NH Asian women when compared with NH White women (adjusted odds ratio (aOR) 2.79; 95% confidence interval (CI) 2.75-2.83), and were also increased in the NH AIAN/NHOPI women (aOR 1.61; 95% CI 1.55-1.67), NH Mixed women (aOR 1.08; 95% CI 1.05-1.11), and Hispanic women (aOR 1.16; 95% CI 1.15-1.17), but decreased in NH Black women (aOR 0.76; 95% CI 0.75-0.77).

Conclusion

Overall, our study found that GDM is associated with the race/ethnicity of mothers. NH Asian and NH AIAN/NHOPI women had the highest rates of GDM when compared with their NH White counterparts. Future research should examine subgroups within larger race categories to better understand the nuances of their experiences and other GDM risk factors, such as the psychosocial experiences of racism and discrimination.

## Introduction

Racial disparities are prevalent in reproductive health outcomes [1]. Gestational diabetes mellitus (GDM) can have long-term negative health consequences, including increased risk for both the mother and child to develop type 2 diabetes mellitus (T2DM) later in life [2]. However, a systematic review and meta-analysis found that screening rates for T2DM post-pregnancy are low [3]. Furthermore, even though Black women with GDM are more likely than their non-Hispanic (NH) White counterparts to develop diabetes mellitus after pregnancy, screening rates for Black women are lower than those of other racial groups [3].

Recognizing the potential long-term impacts of GDM is essential to understanding why racial disparities must be addressed. Patients with GDM have an increased risk of cardiovascular disease in the mother, along with macrosomia and its associated complications in the child [2]. Studies have also found that both pregestational diabetes and GDM may lead to an increased risk of stillbirths [4]. In a population-based retrospective cohort study that examined national birth and death certificate data from 2014 to 2017, there was a larger risk of stillbirth in mothers with GDM when compared to their counterparts who did not have GDM [4]. Therefore, preventing GDM - and providing swift and adequate treatment when it is diagnosed - is an important public health intervention.

Many factors, including the social determinants of health, influence whether an individual develops GDM. For instance, access to affordable, nutritious foods, safe green spaces that are appropriate for exercise, and transportation to primary care and prenatal appointments are all upstream environmental factors that may affect the risk of the patient developing GDM or the management of GDM once diagnosed [5,6]. Additionally, psychosocial stressors and racial discrimination have been found to play a role in promoting poor health outcomes both in general and in reproductive health [6].

Pregnant people of color represent a particularly vulnerable population [6,7]. A 2015 systematic review concluded that management of GDM must be individualized to account for patients’ ethnic differences, as they can significantly affect pregnancy outcomes in patients with GDM [8]. For instance, interventions such as the use of insulin or nutritional counseling should be tailored to individuals rather than presented as a solution to the general population [8]. Even within ethnic groups, those born within their native country may have differing risk profiles from patients born within nations considered to be part of the global north [8]. The impact of health literacy on knowledge of reproductive health and subsequent behavior and outcomes must also be considered, particularly among certain ethnic groups that may have a lower health literacy than others [9]. As the intricacies of these racial disparities are explored, resources and programs that may help address these gaps in reproductive care can be developed. To that end, our study examines if, amongst pregnant women, women of color, when compared with their NH White counterparts, experience higher rates of GDM. Our study aims to assess racial and ethnic disparities in GDM prevalence in the United States.

## Materials and methods

Study design

A retrospective cohort study was conducted based on data acquired through the National Vital Statistics System (NVSS) from the National Center for Health Statistics (NCHS). The NCHS is a federally funded statistics agency. The data we used from the NVSS was collected from birth certificates that were logged in vital statistics centers across each state and Washington, DC.

Population

Inclusion criteria in this study included women who were between the ages of 18 and 54 years, had singleton live births in 2022, and were US residents. Exclusion criteria included women for whom the race/ethnicity or the GDM status was undocumented, as well as women with a prior diagnosis of non-gestational diabetes. Additionally, data on women who had stillbirths were not included in our analysis, as we only considered women who had live births. Although we are aware that non-binary and trans individuals can give birth, since the data focuses on cisgender women specifically, our study focused on their experiences.

Variables

The independent variable was race, defined as NH White, NH Black, NH American Indian or Alaskan Native/Native Hawaiian or Other Pacific Islander (AIAN/NHOPI), NH Asian, NH Mixed, and Hispanic.

The dependent variable or outcome was gestational diabetes, defined by yes, no, unknown/not stated (RF_GDIAB). Potential confounders that were examined included maternal age, BMI, maternal education, payment source for delivery, use of the Women, Infants, & Children (WIC) Program, prenatal care assessed via the Kotelchuck Index, pre-pregnancy smoking, and pre-pregnancy hypertension. Maternal age was divided into the following groups: 18-19, 20-24, 25-29, 30-34, 35-39, and 40-54. BMI was defined as underweight (<18.5), normal (18.5-24.9), overweight (25.0-29.9), obesity I (35.0-34.9), obesity II (35.0-39.9), and extreme obesity III (≥ 40.0). Maternal education was categorized into High School No Diploma, High School, Some College/Associate's, Bachelor's, and Master's/Professional. Payment source for delivery was defined as Medicaid, private, self, or other. The Kotelchuck Index was divided into inadequate (received less than 50% of expected prenatal visits), intermediate (50%-79%), adequate (80%-109%), and adequate plus (110% or more). Use of WIC, pre-pregnancy smoking, and pre-pregnancy hypertension were all defined as Yes or No.

Statistical analysis

Initially, an evaluation of the sample's baseline characteristics was performed. Subsequently, bivariate analyses were conducted to ascertain any association between the exposure and baseline characteristics/potential confounders, as well as between the exposure/baseline characteristics and the outcome. Chi-square test was employed for categorical variables. We also obtained the odds ratio with 95% confidence intervals (CIs); p-values <0.05 were considered statistically significant. Finally, the study included a binary logistic regression to control for confounders and determine any existing associations between the exposure and the outcome.

## Results

**Figure 1:**
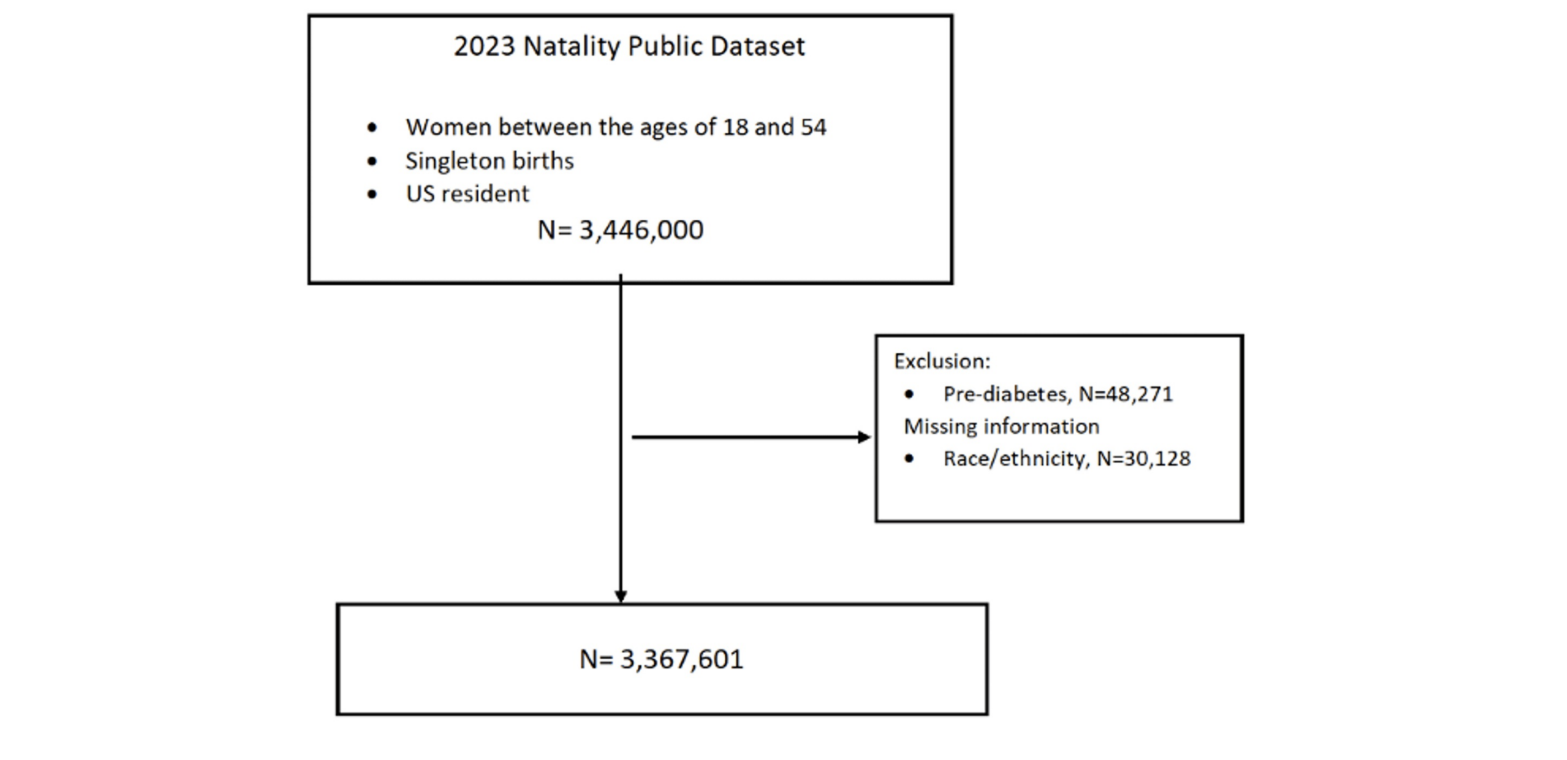
Flowchart of the inclusion and exclusion of participants

In our study, the overall number of patients with singleton pregnancy in the database was 3,446,000. However, 48,271 women were excluded due to pre-pregnancy diabetes, and 30,128 because of missing data regarding race/ethnicity (Figure 1). Thus, a total of 3,367,601 women were included in the analysis (Figure 1). Most women included were NH White women (50.5%), followed by Hispanic women (26.5%), NH Black women (13.5%), and NH Asian women (6.1%).

The overall distribution of baseline characteristics of the sample by race/ethnicity is displayed in Table 1. When looking at demographics, compared to NH White women and NH Asian women, pregnant women who were Hispanic, NH Black, or NH AIAN/NHOPI were significantly younger and had a higher frequency of BMI as overweight and above. In terms of social factors, NH AIAN/NHOPI, NH Black, and Hispanic women more frequently had Medicaid as a source of health coverage, while NH Asian and NH White women had the highest frequency of private insurance. Additionally, NH AIAN/NHOPI and Hispanic women had lower degrees of education, whereas NH Asian women, followed by NH White women, had the highest degrees. Finally, Hispanic, NH Black, and NH AIAN/NHOPI women had a higher frequency of use of WIC and of inadequate prenatal care, as measured by the Kotelchuck Index. Considering chronic conditions, NH Asian women also had the lowest rate of pre-pregnancy smoking, followed by Hispanic women, whereas NH AIAN/NHOPI women had the highest rate. Finally, NH Black women had the highest rate of pre-pregnancy hypertension in comparison to NH Asian and Hispanic women, who had the least.

**Table 1:** Baseline characteristics of the sample of US pregnant women by race/ethnicity NH: Non-Hispanic; AN/NHOPI: American Indian or Alaskan Native/Native Hawaiian or Other Pacific Islander The data are sourced from reference [11].

Characteristics	NH White Women (N=1,700,599)	NH Black Women (N=453,593)	NH AN/NHOPI Women (N=32,018)	NH Asian Women (N=206,760)	NH Mixed Women (N=83,201)	Hispanic Women (N=891,430)	Chi-square	p-value
	%	%	%	%	%	%		
Age (Years)								
18-19	2.1	4.4	5.2	0.4	4.7	4.6	1.2E+05	<0.001
20-24	15.0	21.7	26.2	4.5	23.6	22.7		
25-29	28.3	27.3	29.3	19.2	29.4	29.3		
30-34	33.4	27.1	24.3	41.2	25.9	25.7		
35-39	17.5	15.2	12.3	28.0	13.2	13.9		
40-54	3.7	4.4	2.9	6.9	3.2	3.8		
Body Mass Index (BMI)								
Underweight	2.7	2.7	1.9	4.9	3.2	2.0	1.1E+05	<0.001
Normal	41.9	28.6	25.7	55.0	36.3	31.1		
Overweight	26.5	27.0	26.6	27.0	26.5	31.4		
Obesity I	15.2	20.2	22.5	9.7	17.1	20.4		
Obesity II	7.9	11.6	13.6	2.6	9.5	9.4		
Extreme Obesity III	5.9	10.0	9.8	0.8	7.5	5.6		
Source of Payment								
Medicaid	27.3	64.0	63.2	22.0	47.0	58.5	4.3E+05	<0.001
Private	65.4	30.8	25.3	72.5	45.7	31.8		
Self	4.0	2.4	3.2	2.8	2.5	6.7		
Other	3.3	2.9	8.3	2.8	4.8	3.0		
Education								
HS No Diploma	5.8	10.1	17.9	4.8	9.3	21.0	4.8E+05	<0.001
High School	21.4	38.8	40.2	11.3	29.0	35.3		
Some College/Associate's	25.7	30.3	30.5	14.3	32.0	25.7		
Bachelors	29.1	13.3	8.2	35.4	19.2	12.8		
Masters/Professional	18.0	7.6	3.3	34.2	10.5	5.3		
Women, Infants, & Children (WIC)	19.2	46.0	43.3	16.8	34.0	49.3	3.2E+05	<0.001
Kotelchuck Index [10]								
Inadequate	10.6	22.8	30.8	11.4	16.2	20.5	9.4E+04	<0.001
Intermediate	4.4	6.9	8.2	6.3	6.2	6.6		
Adequate	29.4	25.4	24.6	33.1	28.6	28.5		
Adequate Plus	55.7	45.0	36.5	49.2	49.0	44.4		
Pre-pregnancy Smoking	5.7	3.8	10.0	0.6	7.0	1.4	4.0E+04	<0.001
Pre-pregnancy Hypertension	2.9	5.3	3.3	1.8	3.6	1.9	1.4E+04	<0.001

About 9% of the women included in the sample had GDM. The frequency of GDM according to race/ethnicity and control variables is seen in Table 2. The NH Asian and NH AIAN/NHOPI groups had the greatest frequency of GDM when compared to NH White women and the other groups. The frequency of GDM increased with age and with BMI, and was slightly lower in women with self-pay care. Finally, women with a Kotelchuck index of adequate plus and those with pre-pregnancy hypertension had a higher frequency of GDM than their counterparts. Differences in the frequency of GDM by education, WIC, and pre-pregnancy smoking, although statistically significant, were small in practical terms.

**Table 2:** Baseline characteristics of US pregnant women by gestational diabetes status NH: Non-Hispanic; AIAN/NHOPI: American Indian or Alaskan Native/Native Hawaiian or Other Pacific Islander

Characteristics	Gestational Diabetes				Chi-square	P-value
	Yes (N=281,702)		No (N=3,085,899)			
	N	%	N	%		
Race/Ethnicity						
NH White women	127,385	7.5	1,573,214	92.5	1.9E+04	<0.001
NH Black women	31,126	6.9	422,467	93.1		
NH AIAN/NHOPI women	4,065	12.7	27,953	87.3		
NH Asian women	32,868	15.9	173,892	84.1		
NH Mixed women	6,717	8.1	76,484	91.9		
Hispanic women	79,541	8.9	811,889	91.1		
Age (Years)						
18-19	2,766	2.7	99,421	97.3	3.7E+04	<0.001
20-24	27,542	4.6	565,808	95.4		
25-29	66,873	7.1	872,091	92.9		
30-34	95,674	9.2	939,310	90.8		
35-39	68,042	12.1	495,206	87.9		
40-54	20,805	15.4	114,063	84.6		
Body Mass Index (BMI)						
Underweight	3,273	3.7	84,170	96.3	6.6E+04	<0.001
Normal	59,808	4.8	1,185,760	95.2		
Overweight	72,755	7.9	845,700	92.1		
Obesity I	64,524	11.5	496,075	88.5		
Obesity II	40,225	14.3	241,287	85.7		
Extreme Obesity III	35,423	17.6	165,998	82.4		
Source of Payment						
Medicaid	114,270	8.3	1,257,575	91.7	2.2E+03	<0.001
Private	149,404	8.7	1,568,488	91.3		
Self	7,594	5.2	139,552	94.8		
Other	8,748	8.1	98,718	91.9		
Education						
HS No Diploma	29,814	8.5	319,231	91.5	1.0E+03	<0.001
High School	70,204	7.8	831,939	92.2		
Some College/Associate's	77,966	9.1	780,333	90.9		
Bachelors	61,169	8.1	690,911	91.9		
Masters/Professional	38,836	8.4	423,979	91.6		
Women, Infants, & Children (WIC)						
Yes	91,964	8.8	948,194	91.2	448.4	<0.001
No	186,940	8.2	2,107,268	91.9		
Kotelchuck Index [10]						
Inadequate	33,750	6.7	468,196	93.3	8.1E+03	<0.001
Intermediate	12,434	6.9	168,829	93.1		
Adequate	68,140	7.2	880,325	92.8		
Adequate Plus	162,338	9.7	1,504,628	90.3		
Pre-pregnancy Smoking						
No	269,293	8.3	2,961,155	91.7	86.9	<0.001
Yes	12,409	9.1	124,744	91.0		
Pre-pregnancy Hypertension						
No	263,704	8.1	3,005,642	91.9	1.3E+04	<0.001
Yes	17,998	18.3	80,257	81.7		

As seen in Table 3, the unadjusted odds of GDM were more than twice as high in NH Asian women when compared with NH White women (odds ratio (OR) 2.33; 95% CI 2.30- 2.37). After adjusting for confounders, the difference persisted (adjusted odds ratio (aOR) 2.79; 95% CI 2.75-2.83). The unadjusted odds of GDM were also increased in the NH AIAN/NHOPI women (OR 1.80; 95% CI 1.74-1.86), NH Mixed women (OR 1.08; 95% CI 1.06-1.11), and Hispanic women (OR 1.21; 95% CI 1.20-1.22). All associations remained significant after adjusting for confounders (NH AIAN/NHOPI aOR 1.61; 95% CI 1.55-1.67, NH Mixed aOR 1.08; 95% CI 1.05-1.11, and Hispanic aOR 1.16; 95% CI 1.15-1.17). On the contrary, the odds of GDM in NH Black women were lower than in the reference group both before (OR 0.91; 95% CI 0.90-0.92) and after adjustment (aOR 0.76; 95% CI 0.75-0.77). Incidental findings include an increase in the odds of developing GDM as maternal age and BMI increase. Additional incidental findings include that those who have pre-pregnancy hypertension, use WIC, and receive adequate plus care according to the Kotelchuck Index also have increased odds of being diagnosed with GDM.

**Table 3:** Unadjusted and adjusted associations between race/ethnicity and gestational diabetes in US pregnant women AIAN/NHOPI: American Indian or Alaskan Native/Native Hawaiian or Other Pacific Islander; CI: confidence interval; all p-values < 0.001.

Characteristics	Unadjusted		Adjusted	
	Odds Ratio	95% CI	Odds Ratio	95% Cl
Race/Ethnicity				
Non-Hispanic White women	Reference	-	Reference	-
Non-Hispanic Black women	0.91	0.90-0.92	0.76	0.75-0.77
Non-Hispanic AIAN/NHOPI women	1.80	1.74-1.86	1.61	1.55-1.67
Non-Hispanic Asian women	2.33	2.30-2.37	2.79	2.75-2.83
Non-Hispanic Mixed women	1.08	1.06-1.11	1.08	1.05-1.11
Hispanic women	1.21	1.20-1.22	1.16	1.15-1.17
Age (Years)				
18-19	0.36	0.35-0.38	0.43	0.41-0.44
20-24	0.63	0.63-0.64	0.65	0.64-0.66
25-29	Reference	-	Reference	-
30-34	1.33	1.31-1.34	1.37	1.36-1.39
35-39	1.79	1.77-1.81	1.84	1.82-1.87
40-54	2.38	2.34-2.42	2.37	2.33-2.41
Body Mass Index (BMI)				
Underweight	0.77	0.74-0.80	0.85	0.82-0.88
Normal	Reference	-	Reference	-
Overweight	1.71	1.69-1.72	1.70	1.68-1.72
Obesity I	2.58	2.55-2.61	2.63	2.60-2.66
Obesity II	3.31	3.26-3.35	3.44	3.39-3.49
Extreme Obesity III	4.23	4.17-4.29	4.35	4.28-4.42
Source of Payment				
Medicaid	0.95	0.95-0.96	0.99	0.98-1.00
Private	Reference	-	Reference	-
Self	0.57	0.56-0.58	0.68	0.66-0.70
Other	0.93	0.91-0.95	1.02	1.00-1.05
Education				
HS No Diploma	1.11	1.09-1.12	1.10	1.08-1.12
High School	Reference	-	Reference	-
Some College/Associate's	1.18	1.17-1.20	1.00	0.98-1.01
Bachelors	1.05	1.04-1.06	0.85	0.84-0.86
Masters/Professional	1.09	1.07-1.10	0.80	0.79-0.81
Women, Infants, & Children (WIC)	1.09	1.08-1.10	1.11	1.09-1.12
Kotelchuck Index [10]				
Inadequate	0.93	0.92-0.94	0.98	0.96-0.99
Intermediate	0.95	0.93-0.97	0.93	0.92-0.95
Adequate	Reference	-	Reference	-
Adequate Plus	1.39	1.38-1.41	1.35	1.34-1.37
Pre-pregnancy Smoking	1.09	1.07-1.11	1.09	1.07-1.11
Pre-pregnancy Hypertension	2.56	2.51-2.60	1.61	1.58-1.64

The results of the logistic regression analysis to assess if weight was an effect modifier for the association between race and GDM showed that there was minimal change between the weight strata, indicating that BMI is not an effect modifier. For instance, the odds ratio of NH Black women in the original analysis was 0.87, while the odds ratios of NH Black women using the stratified analysis were 0.88 and 0.71 for the low and high BMI strata, respectively.

## Discussion

Overall, our study found that GDM is associated with the race/ethnicity of mothers. NH Asian, NH AIAN/NHOPI, NH Mixed, and Hispanic women all displayed an increased risk of developing GDM when compared to their NH White counterparts. NH Black women had a lower prevalence of GDM compared to NH White women; once adjusting for confounders, it was confirmed that Black women, with an aOR of 0.76, have a 24% decreased risk of developing GDM when compared to the reference group. This may be due to Black women possibly having lower GDM testing rates or higher rates of pre-existing diabetes.

Our findings align with previously conducted studies examining the relationship between GDM and race. Similar to our findings, four studies found an increased prevalence of GDM in NH non-White women when compared to their NH White counterparts [9,12-14]. Additionally, another study found that NH Asian women, the subgroup of Asian Indian women in particular, had the highest prevalence of GDM [14]. Our study showed NH Asian women to have 179% higher odds of GDM than NH White women. While several studies have assessed these groups, some have not included these populations due to a lack of data on NH Asian and NH AIAN/NHOPI populations. Since our study had sufficient data to examine these two groups, we were able to confirm limited previous findings. While one previous study grouped Asian and Pacific Islander groups together, others were able to examine Asian subgroups such as Asian Indian, Filipino, Japanese, etc. [6,12]. Of note, the study, which stratified the category of Asian, found differences in the prevalence of GDM among these subgroups, with Asian Indians having the highest, and Japanese having the lowest prevalence of GDM [12].

Other studies similarly found the Hispanic and NH Asian/Pacific Islander groups to have the highest prevalence of GDM [6,12]. Additionally, in line with the results of our study, prior studies found NH Black groups to have a slightly lower prevalence of GDM than NH White reference groups [6,12]. One study found the lowest rates of GDM to be among US-born and foreign-born White women, while the highest rates were found in foreign-born Asian/PI/Other, foreign-born Black, and foreign-born Hispanic women [6]. Although we were not able to assess racial discrimination as a possible confounder, the study by Erbetta et al. found a positive association between racial discrimination and GDM [6].

Considering incidental findings, our study found that NH Black women, NH AIAN/NHOPI, NH Mixed, and Hispanic women had a younger maternal age, along with lower education levels, and higher use of WIC and Medicaid. These notable findings indicate an evident need for further exploration of the social determinants of health, as they may be the key to understanding the increased prevalence of GDM seen in most of these communities. Proxies for socioeconomic status in our study were WIC and the payment source of delivery. Patients who qualify for WIC and use Medicaid were considered to be of lower socioeconomic status. Since the database did not contain a category for uninsured, it was assumed that they were accounted for in the self-pay and other categories. However, since this categorization was not clear in the database, our study considered Medicaid to be the marker for lower socioeconomic status. These are important considerations that can help draw a conclusion that a lower socioeconomic status is associated with increased chances of developing GDM. This aligns with the results of a prior study that shows that financial stressors predict a higher risk of GDM [6].

Our study aligns with previous research that found an older maternal age during pregnancy to be associated with a higher risk of GDM [12,13]. After adjusting for confounders, our study also found that the odds for developing GDM decreased as the education level increased and that patients on WIC were found to have an 11% increased chance of GDM, in contrast to patients not enrolled in WIC. The Kotelchuck Index is used to assess levels of prenatal care. Patients belonging to the adequate plus category typically have chronic medical needs and thus require additional care. Our results highlight that those in the adequate plus category have a 35% increased chance of having GDM than those in the adequate group, which is an expected finding given the additional medical needs of people with GDM. Our findings also show an association between GDM and BMI, hypertension, and smoking. This association aligns with previous studies that had similar findings [13-15]. Additionally, in investigating the possibility of BMI being an effect modifier of the association between race and GDM in our study, we conducted an additional statistical analysis in which we stratified the data by obesity. We can conclude that BMI is not an effect modifier after determining a minimal change between the strata.

Given that the data used came from the NVSS and was collected by standard protocols, we can be assured that the data is reliable. Since the data was collected from birth certificates, there is a low risk of recall bias. Nonetheless, there may be recall bias related to information that was self-reported and not obtained from the medical record. The large sample size consisting of women throughout the country is one of the greatest strengths in this study. Limitations of this study include the fact that our study focused on singleton pregnancies and those who are residents of the US. The association between race/ethnicity and GDM might vary if these variables are also considered. For instance, those who are undocumented residents of the US may face food insecurity and inadequate access to healthcare, social determinants of health that can ultimately contribute to a diagnosis of GDM. Additionally, while we considered several potential confounders, there were several that were not accounted for due to a lack of data in the NVSS database, including experiences of racial discrimination and state of residence.

Our study includes data from live births and does not account for the experiences of mothers who had stillbirths. Since GDM is associated with an increased risk of stillbirth, it is important to consider this variable in future studies [16]. Our study also does not account for teen pregnancies or for individuals with pre-pregnancy diabetes. These may be important to explore when understanding why, in our study, NH Black mothers in particular had a lower aOR when compared to their NH White counterparts.

The results of this study can be used to fill in gaps in care so that maternal health disparities can be mitigated. As the differences in the experiences of each racial group are explored, appropriate interventions can be implemented. Since the dataset consisted of women throughout the country, it is apparent that national policies and practices, including those that govern Medicaid and WIC coverage, must be addressed [17]. Future studies can look at subgroups within larger race categories to better understand the nuances of their experiences. For instance, the experiences of NH AIAN/NHOPI women may differ according to their specific backgrounds. They should also examine other risk factors, such as the psychosocial experiences of racism and discrimination. Studies should also explore possible interventions that may be employed to address health disparities in maternal outcomes. As new research is conducted, evidence-based policies can be implemented that can ultimately improve both maternal health and birth outcomes.

## Conclusions

Overall, our study found that GDM is associated with the race/ethnicity of mothers. NH Asian and NH AIAN/NHOPI women had the highest rates of GDM when compared with their NH White counterparts. Of significance, NH Asian women are more than 1.7x likely to develop GDM, while NH AIAN/NHOPI women have a greater than 60% increased chance of developing GDM in comparison to NH White women. Socioeconomic status, maternal age, BMI, hypertension, and smoking all displayed a notable positive correlation to GDM, conveying them as additional factors to consider that have a strong influence on the risk of developing GDM.
